# Blood-Based Detection of Colorectal Cancer Using Cancer-Specific DNA Methylation Markers

**DOI:** 10.3390/diagnostics11010051

**Published:** 2020-12-31

**Authors:** Nam-Yun Cho, Ji-Won Park, Xianyu Wen, Yun-Joo Shin, Jun-Kyu Kang, Sang-Hyun Song, Hwang-Phill Kim, Tae-You Kim, Jeong Mo Bae, Gyeong Hoon Kang

**Affiliations:** 1 Laboratory of Epigenetics, Cancer Research Institute, Seoul National University College of Medicine, Seoul 03080, Korea; chonamyun@hanmail.net (N.-Y.C.); syj4614@naver.com (Y.-J.S.); jeongmobae@gmail.com (J.M.B.); 2 Department of General Surgery, Seoul National University College of Medicine, Seoul 03030, Korea; sowisdom@gmail.com; 3 Guangdong Provincial Key Laboratory of Colorectal and Pelvic Floor Diseases, The Sixth Affiliated Hospital, Sun Yat-sen University, Guangzhou 510655, China; wenxy29@mail.edu.cn; 4 Department of Molecular Medicine and Biopharmaceutical Sciences, Graduate School of Convergence Science and Technology, Seoul National University, Seoul 03080, Korea; jjunk88@snu.ac.kr (J.-K.K.); song1030@snu.ac.kr (S.-H.S.); kimhp@imbdx.com (H.-P.K.); kimty@snu.ac.kr (T.-Y.K.); 5 Department of Internal Medicine, Seoul National University College of Medicine, Seoul 03080, Korea; 6 Department of Pathology, Cancer Research Institute, Seoul National University College of Medicine, Seoul 03080, Korea

**Keywords:** cfDNA, colorectal cancer, droplet digital PCR, MethyLight, methylation, plasma

## Abstract

Cancer tissues have characteristic DNA methylation profiles compared with their corresponding normal tissues that can be utilized for cancer diagnosis with liquid biopsy. Using a genome-scale DNA methylation approach, we sought to identify a panel of DNA methylation markers specific for cell-free DNA (cfDNA) from patients with colorectal cancer (CRC). By comparing DNA methylomes between CRC and normal mucosal tissues or blood leukocytes, we identified eight cancer-specific methylated loci (*ADGRB1*, *ANKRD13*, *FAM123A*, *GLI3*, *PCDHG*, *PPP1R16B*, *SLIT3*, and *TMEM90B*) and developed a five-marker panel (*FAM123A*, *GLI3*, *PPP1R16B*, *SLIT3*, and *TMEM90B*) that detected CRC in liquid biopsies with a high sensitivity and specificity with a droplet digital MethyLight assay. In a set of cfDNA samples from CRC patients (*n* = 117) and healthy volunteers (*n* = 60), a panel of five markers on the platform of the droplet digital MethyLight assay detected stages I–III and stage IV CRCs with sensitivities of 45.9% and 95.7%, respectively, and a specificity of 95.0%. The number of detected markers was correlated with the cancer stage, perineural invasion, lymphatic emboli, and venous invasion. Our five-marker panel with the droplet digital MethyLight assay showed a high sensitivity and specificity for the detection of CRC with cfDNA samples from patients with metastatic CRC.

## 1. Introduction

Liquid biopsies are noninvasive tests that detect fragments of DNA or cells circulating in the blood. Blood samples can be collected without a significant risk of causing harm when trying to reach affected organs that are difficult to access. Cell-free DNA (cfDNA) comes from healthy, inflamed, or cancerous tissue as a result of apoptosis or necrosis. These DNA segments are approximately 170 bp in length on average and have a half-life of approximately 2 h [1,2]. Circulating tumor DNA (ctDNA) refers to tumor cell-derived cfDNA and comprises a minor portion of cfDNA in the blood [3]. Liquid biopsy studies utilize properties of ctDNA, including tumor-specific mutations or copy number alterations, and provide information for early detection, monitoring treatment responses, and recurrence screening.

DNA methylation occurs on the cytosine residue in the context of CpG dinucleotides; there are approximately 2.8 million CpG dinucleotides in the human genome [4]. Although up to 80% of CpG dinucleotides are methylated, CpG sites are differentially methylated; in normal cells, CpG sites located in promoter CpG islands are usually protected from methylation, whereas CpG sites outside of promoter CpG islands are generally methylated. However, cancer cells tend to undergo the opposite methylation changes: the focal hypermethylation of promoter CpG island loci and diffuse genome-wide hypomethylation. These differential methylation patterns between cancer cells and corresponding normal tissues provide a basis for the utilization of DNA methylation as a biomarker for cancer detection. Because DNA methylation signatures of cancer cells are concordant between primary cancer tissue and paired metastatic cancer tissues [5,6,7], DNA methylation profiles in ctDNA are thought to carry DNA methylation signatures of primary cancer tissues. Furthermore, not only normal cells, but also cancer cells of different tissue types are known to have characteristic DNA methylation profiles [6,8,9,10], and these methylation signatures can be used to identify the cell type or tissue type of origin [6,11].

With DNA methylation data from publicly available data sets (Gene Expression Omnibus (GEO) and The Cancer Genome Atlas (TCGA)) from colorectal tumors, normal colonic tissue samples, healthy blood samples, and tumor samples of different cancer types, the Laird team sought to identify DNA methylation markers with both high methylation levels in colorectal cancer (CRC) tissues and low methylation levels in nonneoplastic mucosal tissues, blood leukocytes, and other tissue types of cancer and found that two DNA methylation markers, *THBD* and *c9orf50* [12], detected CRC in cfDNA from clinical samples with a high sensitivity and specificity. In the present study, we identified new candidate DNA methylation markers for the blood-based detection of CRCs by comparing DNA methylomes of genomic DNA samples from CRC and nonneoplastic colonic tissue samples and peripheral blood leukocytes, which were obtained from publicly available datasets (TCGA and GEO). Using a droplet digital PCR-based MethyLight (ddMethyLight) assay, we assessed whether the number of methylated DNA alleles in cfDNA was proportional to the tumor size in xenografted mice and then compared the sensitivity and specificity for the detection of CRC with cfDNA samples between the newly identified markers and *THBD* and *c9orf50*. We found that the newly identified markers were not superior to *THBD* and c9orf50 for the detection of CRC with cfDNA samples but that a panel of five markers were superior to *THBD* and *c9orf50*.

## 2. Materials and Methods

### 2.1. Blood Samples

Blood samples were obtained from healthy volunteers (*n* = 60) and patients with CRC (*n* = 117). Blood samples were taken from patients with CRC immediately before surgery. Clinicopathological information from CRC patients was retrieved from electronic medical records, including Tumor, Node, Metastasis (TNM) staging, perineural invasion, lymphatic emboli, venous invasion, and history of neoadjuvant therapy. Peripheral blood was collected into EDTA tubes and was used for plasma preparation within 1 hr. Blood samples were centrifuged at 2000× *g* for 10 min, and the plasma was removed without disturbing sedimented cells. The plasma samples were transferred to microcentrifuge tubes and then stored at −20 °C. The frozen plasma samples were thawed and centrifuged at 12,000× *g* for 3 min. The supernatants were used for DNA isolation. Informed consent was obtained from all participants. This study was approved by the Institutional Review of Board of Seoul National University Hospital (IRB No. H-1608-040-784) (11 August 2016) and conducted in compliance with the principles of the Declaration of Helsinki and its later amendments.

### 2.2. Cell Culture and Reagents

Two human CRC cell lines (SNU407 and SW620) were obtained from the Korean Cell Line Bank (Seoul, Korea) and grown in RPMI-1640 medium supplemented with 10% fetal bovine serum and 1% antibiotic solution containing penicillin and streptomycin. Cells were incubated at 37 °C in a humidified atmosphere with 5% CO_2_.

### 2.3. Xenograft Mouse Model

A total of 20 male BALB/c nude mice (6 weeks old) were purchased from Orient Bio. (Gyeonggi-do, Korea), and 10 mice were used for each cell line xenograft experiment. All experimental procedures and animal care were conducted in accordance with the guidelines on the ethical use of animals that were approved by the Institutional Animal Care and Use Committee of Seoul National University Hospital (IACUC approval number: 17-0029-C1A0) (10 November 2017). A total of 1 × 10^6^ viable cells suspended in 0.1 mL of Matrigel (BD Biosciences, San Jose, CA, USA) were injected subcutaneously into the right flank. One, two, and three weeks after the appearance of a xenograft mass at the inoculation site, the mice were euthanized with CO_2_. Peripheral blood samples obtained from xenografted mice were collected in EDTA tubes and subjected to DNA preparation from plasma, as described above.

### 2.4. DNA Extraction and Bisulfite Modification

Plasma DNA was isolated using NucleoSpin Plasma XS (MACHERY-NAGEL GmbH & Co., KG, Duren, Germany) (Supplementary Figure S1). These extractions were performed according to the manufacturer’s protocols. Briefly, 240 μL of thawed plasma was mixed with 4 μg polyadenylic acid (poly(A); Roche Diagnostics, Mannheim, Germany) and 20 μL proteinase K (≥0.4 U/μL) and then incubated at 37 °C for 10 min. After that, the extraction process was carried out according to the manufacturer’s protocol, with 48 μL elution buffer. For 1 mL of thawed plasma, four aliquots of 240 μL were subjected to the cfDNA extraction process. Genomic DNA was extracted from cell lines and peripheral blood leukocytes (*n* = 10) using a QIAamp DNA Mini kit (Qiagen N.V., Hilden, Germany). One microgram of genomic DNA and 60 μL of cfDNA samples were subjected to bisulfite modification using an EZ DNA methylation kit (Zymo Research, CA, USA).

### 2.5. Selection of Target Probes

**Figure 1:**
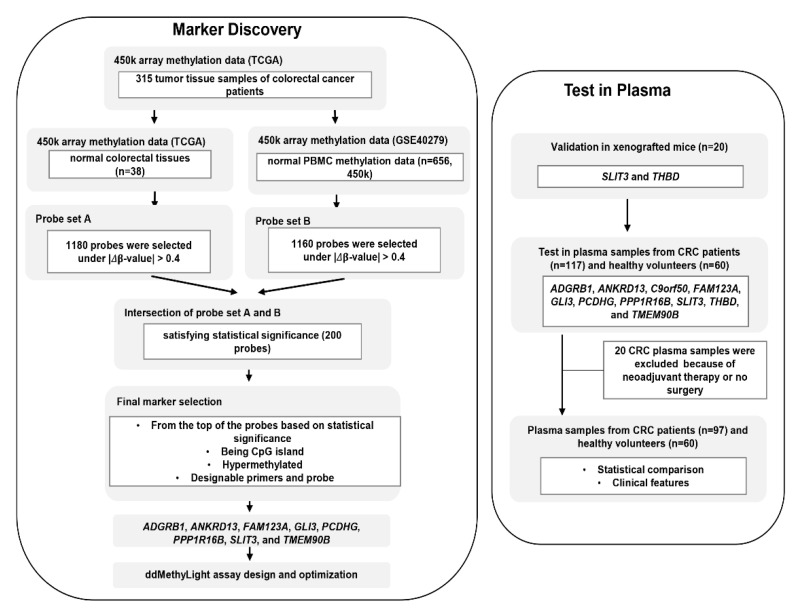
Overview of study workflow from marker discovery to verification in plasma samples. The process of selection of candidate DNA methylation markers and their verification in detection of colorectal cancer (CRC) in cell-free DNA (cfDNA) samples from patients with CRC were schematically represented.

Tissue DNA methylation data (Infinium HumanMethylation450 BeadChip array) for CRC samples that included 315 tumor and 38 paired normal tissues (adjacent normal tissues) were obtained from TCGA. Complete clinical, molecular, and histopathological data sets are available at the TCGA website (https://tcga-data.nci.nih.gov/docs/publications/tcga). DNA methylation data was obtained for 656 blood leukocyte samples from healthy control individuals from a data set used in a methylation study on aging (GSE40279). The differentially methylated probes were selected from the dataset. First, the delta-beta value was calculated by subtracting each mean normal value from the tumor sample value. Second, only probes with an absolute delta-beta value of 0.4 or higher were selected. A total of 1180 probes were selected in this step (probe set A). The above procedure was performed again using the GEO dataset (GSE40279) as normal samples, and 1160 probes were selected (probe set B). Two hundred probes were selected by intersecting probe sets A and B and selecting significant probes (*p* < 0.05) (Figure 1). From the top of the 200 probes based on significance, we selected eight probes that fulfilled the following criteria: (1) hypermethylated in tumors compared with normal tissues and blood cells, (2) interrogating a CpG island locus, and (3) designable primer and probe set for the MethyLight assay.

### 2.6. ddMethyLight Assay

Oligonucleotide sequences for the primers and probes of methylated *ADGRB1*, *ANKRD13*, *C9orf50*, *FAM123A*, *GLI3*, *PCDHG*, *PPP1R16B*, *SLIT3*, *THBD*, and *TMEM90B* are listed in Supplementary Table S1. The probe sequences for methylated *ADGRB1*, *ANKRD13*, *C9orf50*, *FAM123A*, *GLI3*, *PCDHG*, *PPP1R16B*, *SLIT3*, *THBD*, and *TMEM90B* were synthesized with a FAM reporter. The ddMethyLight reaction mixture consisted of 2X ddPCR Supermix for Probes (BioRad Cat #186–3010, Hercules, CA, USA) and oligonucleotide primers and probes (final concentrations of 300 and 100 nmol/L, respectively). The relative amounts of each sample were determined with a C-LESS-C1 assay, which controls the total input amount of DNA in PCR [13]. The C-LESS probe was synthesized with a VIC reporter. Various amounts of bisulfite-converted DNA were used in a final volume of 20 µL. PCR products (20 µL) and droplet generation oil (70 µL) were separately loaded into adjacent wells of a Bio-Rad DG8 disposable droplet generation cartridge (BioRad, Hercules, CA, USA). The samples and oil were combined within the microchannels of the cartridge to generate an emulsion of droplets, which were then pipette-transferred to a 96-well PCR plate. The plate was amplified with the following cycling conditions: 95 °C for 10 min; 45 cycles of 95 °C for 15 s and 60 °C for 1 min; and a 10-min hold at 98 °C. Following amplification, the droplets were read using a 2-color fluorescence reader (QX200 droplet reader, Bio-Rad), which determined how many droplets were positive for methylated *ADGRB1*, *ANKRD13*, *C9orf50*, *FAM123A*, *GLI3*, *PCDHG*, *PPP1R16B*, *SLIT3*, *THBD*, and *TMEM90B* (in FAM) as well as for the control reaction C-LESS-C1 (in VIC). Each PCR plate included no-template-control (NTC) wells, which did not produce an amplification signal. Data analysis was performed using QuantaSoft software version 1.7.4.0917 (BioRad).

### 2.7. Data Analysis of ddMethyLight

An event with a fluorescence amplitude value greater than the set detection threshold was considered a methylation-positive droplet, and methylation-positive droplets were counted for each well. The count of methylation-positive droplets was determined for each marker and cfDNA sample from normal volunteers and cancer patients. The number of markers with one or more methylation-positive droplet(s) and the number of markers with two or more methylation-positive droplets were determined.

### 2.8. Statistical Analysis

Statistical analyses were conducted using SPSS version 25 (IBM, Armonk, NY, USA). To determine whether the number of methylated markers or the number of methylated droplets were normally distributed, we performed a normality test using the Shapiro–Wilk test and determined skewness and kurtosis z-values, which revealed that the number of methylated markers or methylated droplets were not normally distributed. Thus, the Mann–Whitney test and the Kruskal–Wallis test were used to compare two independent groups and more than two independent groups, respectively. Receiver operating characteristic (ROC) analysis was performed, and the area under a given ROC curve (AUC) was obtained.

## 3. Results

The overall workflow of the present study is schematically represented in Figure 1. For the DNA methylation markers selected based on the criteria described above, we designed MethyLight primers and probes for eight methylated markers (*ADGRB1*, *ANKRD13*, *FAM123A*, *GLI3*, *PCDHG*, *PPP1R16B*, *SLIT3*, and *TMEM90B*). To examine whether these eight DNA methylation markers were comparable to the Laird team’s DNA-methylation markers *THBD* and *c9orf50* for the detection of CRCs with plasma cfDNA samples, we analyzed the performance of these ten methylation markers.

### 3.1. Proportional Increase in Methylated DNA Alleles in cfDNA Samples from Xenografted Mice

cfDNA samples were obtained from xenografted mice that were inoculated with one of two human colorectal cancer cell lines, SW620 and SNU407, which were analyzed for the methylation status of *THBD* and *SLIT3* using a MethyLight assay. We analyzed cfDNA samples for the methylation status of *THBD* and *SLIT3* using ddMethyLight. cfDNA samples from mice that were not inoculated with cell lines were negative for methylated *THBD* and *SLIT3*. As the duration from inoculation increased from 0 to 2 weeks, cfDNA samples tended to show an increasing number of droplets that were positive for methylated DNA alleles (*THBD* and *SLIT3*) (Supplementary Figure S1).

### 3.2. Sensitivity and Specificity of Ten DNA Methylation Markers for the Detection of CRC with cfDNA Samples

To compare the performance of the ten DNA methylation markers, we analyzed cfDNA samples from patients with CRC (*n* = 117) and healthy volunteers (*n* = 60) for the methylation statuses of 10 DNA methylation markers using ddMethyLight. The AUC values of 10 individual markers are summarized in Supplementary Table S2. When individual markers were considered positive if any methylated droplet was detected, individual methylation markers showed a sensitivity from 22.2 to 52.1% and specificity from 71.7 to 96.7%. *PPP1R16B*, *PCDHG*, and *TMEM90B* displayed a sensitivity ≥ 57.0% and specificity ≥ 81.7%. When two or more methylation-positive droplets were considered the cutoff for positivity, individual markers exhibited a sensitivity from 10.3 to 23.1% and specificity from 95.0 to 100%. *GLI3*, *PPP1R16B*, and *TMEM90B* showed a sensitivity ≥ 21.4% and specificity ≥ 96.7%. The eight markers were comparable to *THBD1* and *c9orf50* for both sensitivity and specificity. The sensitivity and specificity of 10 individual markers are summarized in Table 1.

### 3.3. Panels of DNA Methylation Markers and the Comparison of Their Sensitivity

To increase the sensitivity and specificity for the detection of CRC with cfDNA samples, combinations of the eight markers that did not include *THBD* and *c9orf50* were examined. For the combination, markers with ≥1 and zero methylation-positive droplet(s) were scored as “1” and “0”, respectively. Of the possible combinations that included from two to eight markers, six markers, *ANKRD13*, *FAM123A*, *GLI3*, *PCDHG*, *PPP1R16B*, and *TMEM90B*, showed the highest AUC (Table 2). When the sum of score(s) ≥2 was regarded as the cutoff for positivity, the six-marker panel exhibited a sensitivity of 74.4% and specificity of 85.7% for the detection of CRC with cfDNA samples. However, because the panel showed a specificity <86%, we sought to find a panel that showed a specificity ≥ 95% and sensitivity ≥ 50%. A five-marker panel, *FAM123A*, *GLI3*, *PPP1R16B*, *SLIT3*, and *TMEM90B*, displayed a sensitivity of 57.3% and specificity of 95.0%. When markers with ≥2 and <2 methylation-positive droplet(s) were scored as “1” and “0”, respectively, and the sum of score(s) ≥1 was regarded as the cutoff for positivity, the five-marker and six-marker panels displayed a sensitivity of 47.0% and 59.0% and specificity of 96.7% and 91.7%, respectively.

### 3.4. Detection of Methylated DNA in cfDNA and Its Relationship with Clinicopathological Features

**Figure 2:**
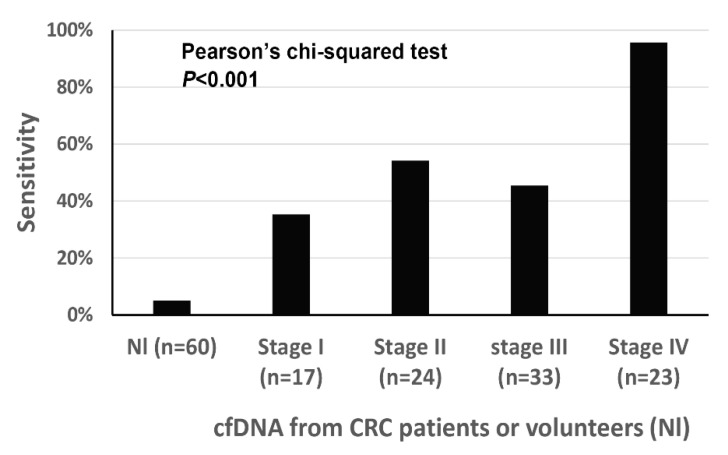
The sensitivity of the five-marker panel for the detection of colorectal cancer (CRC) in cfDNA samples from patients with CRC.

When excluding cases (*n* = 20) in which (1) blood samples were obtained after neoadjuvant chemoradiation for rectal cancer or (2) surgery was not performed, clinicopathological findings were correlated with ddMethyLight results of five markers. Demographical findings of CRCs (*n* = 97) were summarized in Table 3. Of the 97 cases, 94 cases were classified as adenocarcinoma, not otherwise specified, two cases as mucinous adenocarcinoma, and the remaining one as signet ring cell carcinoma. When the cutoff was set at ≥2 markers with ≥1 methylation-positive droplet(s), the five-marker panel displayed a sensitivity of 57.7% and specificity of 95.0% for tumor detection. The five-marker panel showed sensitivities of 35.3%, 54.2%, 45.5%, and 95.7% for stages I, II, III, and IV, respectively (Figure 2). The number of DNA methylation markers with ≥1 methylation-positive droplet(s) was significantly higher in CRCs with distant metastasis than in CRCs without distant metastasis, in CRCs with nodal metastasis than in CRCs with no nodal metastasis, in CRCs with a higher T category than in CRCs with lower T category, in CRCs with lymphatic emboli than in CRCs without lymphatic emboli, in CRCs with venous invasion than in CRCs without venous invasion, and in CRCs with perineural invasion than in CRCs without perineural invasion (Table 3).

## 4. Discussion

For the detection of methylation markers in cfDNA samples, the Laird team’s study applied digital MethyLight PCR in which bisulfite-modified DNA samples were diluted, distributed in 96 PCR wells, and then subjected to a MethyLight assay [12]. The digital MethyLight assay was significantly superior to the classic MethyLight assay for detecting a small number of methylated DNA molecules in the background of a large excess of unmethylated DNA molecules, for example, plasma cfDNA samples [13]. However, because the digital MethyLight assay must be run on, at least, a 96-well plate for each sample and DNA methylation marker, its labor- and reagent-intensive nature has led to the development of droplet digital PCR-based MethyLight (ddMethyLight), which has been demonstrated to have a 20-fold lower limit of detection than conventional MethyLight [14]. Although Yu et al. applied droplet digital PCR to detect methylated alleles for the first time, they did not analyze the performance of ddMethyLight for cfDNA samples [14]. A recent study by Jensen et al. demonstrated the capability of the ddMethyLight assay to detect rare methylated DNA alleles in cfDNA samples [15].

In the present study, we examined whether the number of methylated DNA alleles in cfDNA, as assessed by ddMethyLight, reflected the tumor extent in a xenograft mouse model. We found that the number of methylation-positive droplets increased during the two weeks after inoculation with the cell lines, which indicates that the number of methylation-positive droplets detected by ddMethyLight might be used to assess the tumor extent. However, in plasma samples of patients with CRC, the number of methylation-positive droplets was not different in cfDNA samples among patients with stages I–III CRC (Supplementary Table S3) and the percentage of patients who had tested positive for each marker was not different among stages I, II, and III (Supplementary Table S4). Instead, the number of methylation-positive droplets was significantly higher in cfDNA samples from patients with stage IV CRC than in those from patients with stages I–III CRC and the percentage of patients who were positive for the methylated droplet was higher in stage IV than in stages I–III (Supplementary Tables S3 and S4). In addition to the number of methylation-positive droplet(s) for individual markers, the number of markers with ≥1 methylation-positive droplet(s) was significantly higher in stage IV CRCs than in stages I–III CRCs (Supplementary Figure S2). However, the number of markers with ≥1 methylation-positive droplet(s) was not significantly different among stages I–III CRCs. A five-marker panel with a cutoff value of ≥2 markers with ≥1 methylation-positive droplet(s) showed sensitivities of 35.3%, 54.2%, 45.5%, and 95.7% for stages I, II, III, and IV CRCs, respectively, at a specificity of 95.0%. Our findings suggest that the blood-based detection of CRC using multiple methylation markers might be useful for predicting the presence of metastatic CRC rather than for diagnosing localized CRCs. In contrast, in a recent study by Jensen et al., the ddMethyLight-based detection of a three-marker panel in cfDNA samples, called the TriMeth test, exhibited sensitivities of 80.5%, 84.6%, 89.1%, and 88.2% for stages I, II, III, and IV CRCs, respectively, at a specificity of 99%, which indicates that the three-marker panel with the ddMethyLight platform enables the detection of early-stage CRC [15]. The marked difference in the detection of stages I–III CRCs with cfDNA samples between the present study and the Jensen et al. study could be attributed to the difference in the volume of plasma required for ddMethyLight. In the present study, 50 μL of plasma was used for each ddMethyLight assay (Supplementary Figure S3), whereas in the Jensen et al. study, 1.2 to 5.4 mL of plasma was utilized for each ddMethyLight assay.

The sampling of a large amount of blood decreases compliance from patients and the merits of liquid biopsy: noninvasiveness and comfort. Thus, the requirement of a large blood volume for the DNA methylation-based detection of CRCs might hinder its clinical application as a screening test or clinical test. In the present study, the five-marker panel test utilized 250 μL of plasma, which is smaller than the 3.5 mL of plasma required for the *SEPT9* assay, called the Epi proColon test. The overall sensitivity of the five-marker panel was 57.3% at a 95.0% specificity with the ddMethyLight assay, which is comparable to the sensitivity and specificity of the Epi proColon test in a large-scale cross-sectional study (48.2% and 91.5%, respectively) [16]. Recently, Luo et al. demonstrated that the methylation testing of a single marker (cg1067383300) on the ddMethyLight platform identified 90.5% of participants with stages I–III CRC at a specificity of 86.8%. However, this single-marker test utilized 1.5 mL of plasma [17].

The present study was limited by the lack of data regarding the tissue-type specificity of the five-marker panel. At present, it is unknown whether the five-marker panel can differentiate CRC from cancers of other tissue types. When referring to Infinium-based DNA methylation datasets that are publicly available on TCGA, CpG sites located in the 5’ promoter regions of the genes (*FAM123A*, *GLI3*, *PPP1R16B*, *SLIT3*, and *TMEM90B*) harbor a high level of methylation in CRCs but low levels of methylation in most other tissue types of cancer (Supplementary Figure S4). Of 32 cancer types, gastric carcinoma shows a high level of methylation in the five genes, and diffuse large B cell lymphoma shows a high level of methylation in four genes (not including *PPP1R16B*). The candidate marker approach is unlikely to achieve the simultaneous detection and localization of tumors in liquid biopsy. A recent study by Liu et al. demonstrated that multiple tissue types of cancer across all stages could be detected and localized at a high specificity through the cfDNA sequencing of >100,000 informative methylation regions, although 10 mL of plasma was utilized for capture-based bisulfite sequencing [18,19].

In summary, we developed a five-marker panel that detected CRC with a limited volume of blood on the ddMethyLight platform. The marker panel detected stages I–III CRCs and stage IV CRCs at a sensitivity of 45.9% and 95.7%, respectively, and at a specificity of 95.0%. Because of the low sensitivity in detection of stages I–III CRCs, the marker panel might serve as an ancillary tool in tumor detection before surgery and monitoring for tumor recurrence after surgery. The effectiveness of the marker panel should be validated in an independent set of blood samples from CRC patients and normal volunteers.

## Figures and Tables

**Table 1:** Sensitivity and specificity of each marker according to the different criteria of positivity.

	Cut-off for Positivity Set at 1 Methylated Droplet		Cut-off for Positivity Set at 2 Methylated Droplets	
	Sensitivity (%)	Specificity (%)	Sensitivity (%)	Specificity (%)
*TMEM90B*	52.1	81.7	22.2	100.0
*PCDHG*	48.7	81.7	20.5	95.0
*PPP1R16B*	47.0	86.7	23.1	96.7
*ADGRB1*	47.0	71.7	23.9	95.0
*GLI3*	43.6	93.3	21.4	100.0
*ANKRD13B*	41.0	88.3	20.5	98.3
*THBD*	39.3	78.3	20.5	100.0
*c9orf50*	31.6	85.0	17.1	96.7
*FAM123A*	23.1	96.7	10.3	100.0
*SLIT3*	22.2	95.0	10.3	100.0

**Table 2:** Summary of combinatory panels of methylation markers with high area under curves (AUCs).

No. of Markers	Sum of Markers with ≥ 1 Methylated Droplet(s)								AUC	95% C.I.	
										Lower	Upper
6		*ANKRD13*	*FAM123A*	*GLI3*	*PCDHG*	*PPP1R16B*		*TMEM90B*	0.866	0.814	0.917
8	*ADGRB1*	*ANKRD13*	*FAM123A*	*GLI3*	*PCDHG*	*PPP1R16B*	*SLIT3*	*TMEM90B*	0.862	0.809	0.915
7	*ADGRB1*	*ANKRD13*	*FAM123A*	*GLI3*	*PCDHG*	*PPP1R16B*		*TMEM90B*	0.860	0.807	0.913
5			*FAM123A*	*GLI3*		*PPP1R16B*	*SLIT3*	*TMEM90B*	0.839	0.782	0.897
6	*ADGRB1*		*FAM123A*	*GLI3*		*PPP1R16B*	*SLIT3*	*TMEM90B*	0.828	0.769	0.888
5	*ADGRB1*		*FAM123A*	*GLI3*		*PPP1R16B*	*SLIT3*		0.823	0.762	0.884
3				*GLI3*		*PPP1R16B*		*TMEM90B*	0.822	0.761	0.884

**Table 3:** The number of methylated markers in cfDNA samples in relation to clinicopathological features of colorectal cancer patients (*n* = 97).

		n	Mean	S.D.	*p*-Value **
Tumor subsite	Right colon	15	1.73	1.438	0.675
	Left colon	63	2.02	1.314	
	Rectum	19	1.89	1.243	
Differentiation *	WD	8	1.13	1.126	0.051
	MD	79	1.95	1.300	
	PD	10	2.55	1.265	
Lymphatic emboli	Absent	57	1.60	1.193	0.001
	Present	40	2.45	1.319	
Venous invasion	Absent	77	1.71	1.037	0.011
	Present	20	2.85	1.814	
Perineural invasion	Absent	60	1.68	1.157	0.028
	Present	37	2.38	1.441	
T category	1	8	1.13	0.991	0.001
	2	17	1.41	0.870	
	3	53	1.87	1.225	
	4	19	3.00	1.414	
N category	0	44	1.48	0.849	0.007
	1	33	2.24	1.480	
	2	20	2.50	1.539	
M category	0	74	1.53	0.996	<0.001
	1	23	3.30	1.295	
Stage	I	17	1.24	0.831	<0.001
	II	24	1.67	0.816	
	III	33	1.58	1.173	
	IV	23	3.30	1.295	

* WD, well-differentiated; MD, moderately differentiated; PD, poorly differentiated. ** For the comparison between two groups or three or more groups, the Mann–Whitney test and Kruskal–Wallis test were used, respectively.

## Data Availability

The data presented in this study are available in the manuscript and supplementary materials.

## Supplementary Materials

Supplementary materials can be found at https://www.mdpi.com/2075-4418/11/1/51/s1, Figure S1. The number of methylated droplets (A, *THBD*; B, *SLIT3*) was determined in cfDNA samples from xenografted mice that were inoculated with the SNU-407 or SW620 cell line, Figure S2. The number of markers that showed ≥1 methylation-positive droplet(s) in cfDNA samples from healthy volunteers (Nl) and patients with colorectal cancer, Figure S3. For each droplet digital MethyLight reaction, 6 µL of modified DNA was used, which was equivalent to 50 µL of plasma, Figure S4. Box plots of the beta values of five methylation markers in various tissue types of human cancers (TCGA data), Table S1. Oligonucleotide sequences of primers and probes of DNA methylation markers, Table S2. AUC values of 10 methylation markers, Table S3. The number of methylated droplets in cfDNA samples -from patients with stage I- IV CRC, Table S4. The percentage of healthy volunteers or CRC patients who have tested positive for each marker at each stage.

## Abbreviations

CRC	Colorectal cancer
cfDNA	Cell-free DNA
ctDNA	Circulating tumor DNA
GEO	Gene Expression Omnibus
TCGA	The Cancer Genome Atlas
ddMethyLight	droplet digital PCR-based MethyLight
