# Supplementary material for: Blood-Based Detection of Colorectal Cancer Using Cancer-Specific DNA Methylation Markers
Source: Diagnostics (Basel). 2020 Dec 31;11(1):51. doi: 10.3390/diagnostics11010051 (PMC7823774; doi:10.3390/diagnostics11010051)
Supplement: Supplementary file 1 [file diagnostics-11-00051-s001.pdf]

A

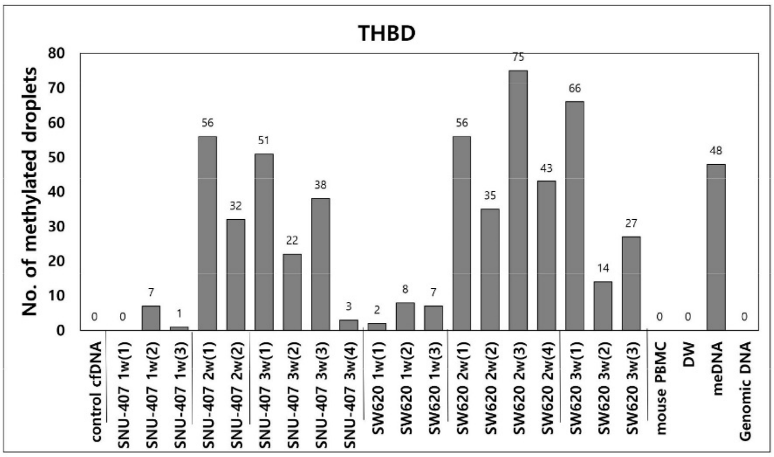

B

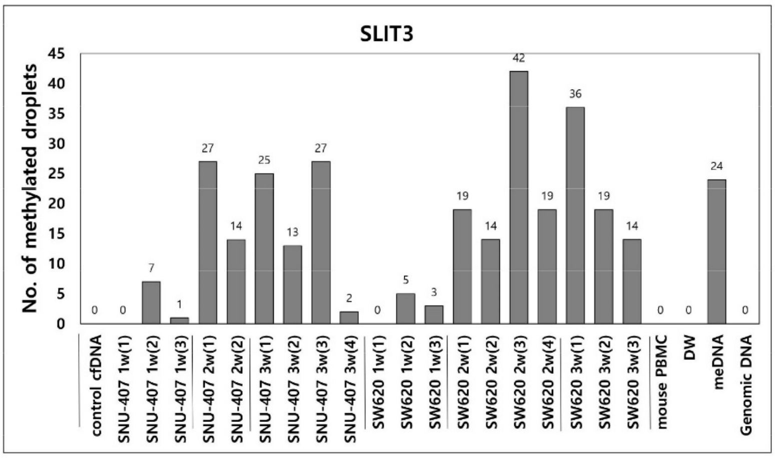

Supplementary Figure S1. The number of methylated droplets (A, *THBD*; B, *SLIT3*) was determined in cfDNA samples from xenografted mice that were inoculated with the SNU-407 or SW620 cell line. Xenografted mice were sacrificed before inoculation and at 1, 2, and 3 weeks after inoculation. PBMCs, bisulfite-treated genomic DNA from peripheral blood mononuclear cells of mouse; DW, distilled water; meDNA, in vitro methylated DNA (purchased from Zymo Research); genomic DNA, genomic DNA from human placental tissue without bisulfite modification.

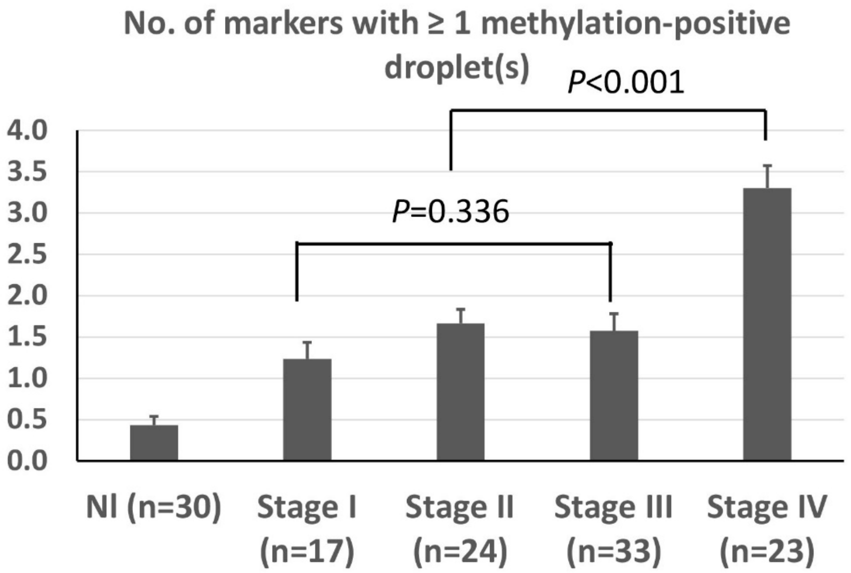

Supplementary Figure S2. The number of markers that showed ≥1 methylation-positive droplet(s) in cfDNA samples from healthy volunteers (Nl) and patients with colorectal cancer.

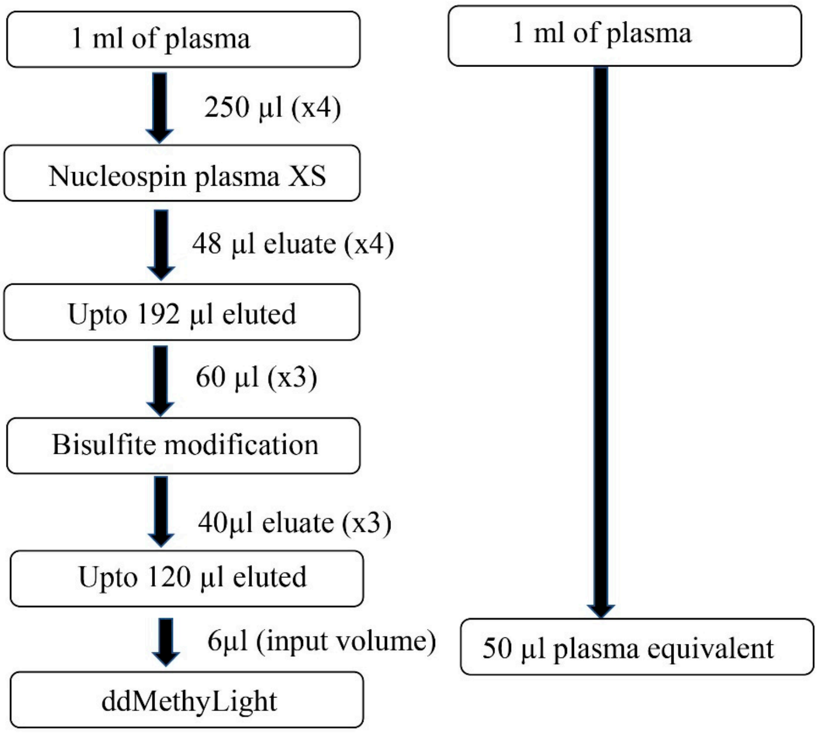

Supplementary Figure S3. For each droplet digital MethyLight reaction, 6 µl of modified DNA was used, which was equivalent to 50 µl of plasma.

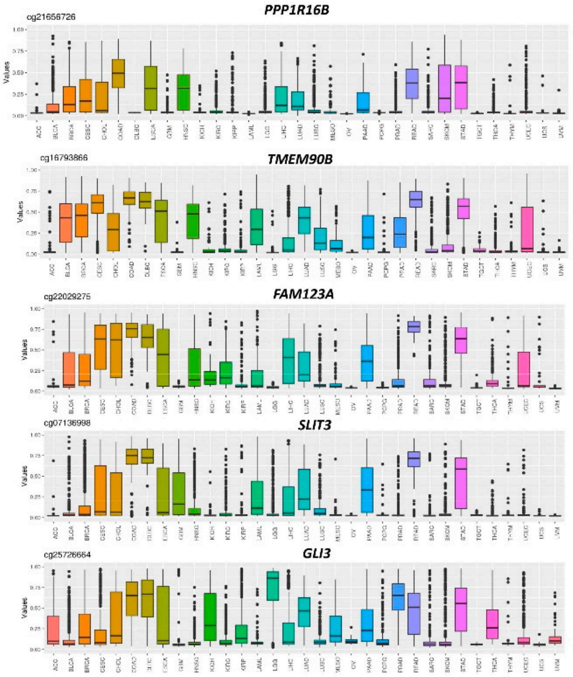

Supplementary Figure S4. Box plots of the beta values of five methylation markers in various tissue types of human cancers (TCGA data).

Supplementary Table S1. Oligonucleotide sequences of primers and probes of DNA methylation markers

Gene	Illumina's Cluster CpG Number	Primers & Probes	Sequence (5'-3')	Amplicon Size (bp)
ADGRB1	cg11758945	ML192-F	AAAATCCCGAAACGCCCGAC	81
		ML192-R	CGTAATAAGTTTGAAAGTAGCGTTAAGG	
		ML192-Pr	6FAM-ACGCCCTCCCTTCGACTCGCTAACTA-BHQ-1	
ANKRD13B	cg14215472	ML191-F	TTTACGTCGTAGCGTTAGTTTGC	100
		ML191-R	CGTCGTAACTCCGAAACACGTA	
		ML191-Pr	6FAM-CCGCCGAAACCTAACCCTAAACTAAACCGA-BHQ-1	
FAM123A	cg22029275	ML185-F	CGAATAACAATAACCTAACGCGAAC	107
		ML185-R	TCGTTGATAGTTTCGTCGTCGT	
		ML185-Pr	6FAM-CACTACTAACGAAAACGACTTATCCCGCGC-BHQ-1	
GLI3	cg25726664	ML183-F	TACGAGTGTTAGGGCGCGTA	88
		ML183-R	AAAACCCGAACGTAATATCACCC	
		ML183-Pr	6FAM-TCTTCTCTCTCCACACCGCCAAAACACC-BHQ-1	
PCDHG	cg04453180	ML190-F	TCCAATTAATACTAACGACGTTAAACGA	95
		ML190-R	CGTATTGTTATTCGCGTTTAGTATTTTGA	
		ML190-Pr	6FAM-CCCAACTCTCTCCGCCAACCTACCTATTC-BHQ-1	
PPP1R16B	cg21656726	ML186-F	GAGCGTCGGGATTTTGTTTCG	92
		ML186-R	CCCCAAAACGAAACCTAAACTCC	
		ML186-Pr	6FAM-AACTAAAATAAACGCGCTCAACCCCATCCG-BHQ-1	
SLIT3	cg07136998	ML166-F	CGTGTTTATTGAATGGAGAGAGCG	79
		ML166-R	AAAACGCGACGACTACGACTA	
		ML166-Pr	6FAM-ACACGAAACGACCGAATAAACTAACCGACG-BHQ1	
TMEM90B	cg16793866	ML182-F	AAATACGCTAACGCCGCGAC	92
		ML182-R	CGGTCGGTGTTTGTTGTATTAAATATTAG	
		ML182-Pr	6FAM-CGAACCGATACCTACGCCTTCCCTAACCA-BHQ-1	

Supplementary Table S2. AUC values of 10 methylation markers.

1 Droplet	AUC	95% C.I.	
		Lower	Upper
GLI3	0.692	0.616	0.768
TMEM90B	0.689	0.613	0.766
PPP1R16B	0.677	0.598	0.755
PCDHG	0.661	0.580	0.741
ANKRD13B	0.656	0.577	0.736
ADGRB1	0.616	0.533	0.700
THBD	0.610	0.528	0.693
FAM123A	0.600	0.517	0.684
c9orf50	0.591	0.506	0.676
SLIT3	0.589	0.504	0.673

Supplementary Table S3. The number of methylated droplets in cfDNA samples form patients with stage I–IV CRC

	**Stage I (n=17)**	**Stage II (n=24)**	**Stage III (n=33)**	**Stage IV (n=23)**	***P*-value (stage I-III)**	***P*-value (stage I-IV)**
FAM123A	0.18	0.17	0.09	29.48	0.595	<0.001
PPP1R16B	0.41	0.46	0.48	74.87	0.755	0.033
TMEM90B	0.35	0.50	0.39	65.96	0.593	<0.001
GLI3	0.42	0.18	0.46	13.43	0.090	0.015
SLIT3	0.12	0.08	0.18	26.13	0.531	<0.001
PCDHG	0.47	0.33	0.48	14.04	0.445	0.005
ANKRD13B	0.35	0.46	0.30	13.61	0.368	0.003
ADGRB1	0.53	0.33	0.36	29.74	0.441	0.006
THBD	0.29	0.33	0.18	87.13	0.412	<0.001
C9orf50	0.47	0.08	0.21	25.13	0.016	<0.001

Supplementary Table S4. The percentage of healthy volunteers or CRC patients who have tested positive for each marker at each stage.

	**Nl (n=60)**	**Stage I (n=17)**	**Stage II (n=24)**	**Stage III (n=33)**	**Stage IV (n=23)**	***P*-value**
FAM123A	3.3%	17.6%	16.7%	9.1%	56.5%	<0.001
PPP1R16B	13.3%	41.2%	45.8%	48.5%	69.6%	<0.001
TMEM90B	18.3%	35.3%	50.0%	39.4%	87.0%	<0.001
GLI3	6.7%	17.6%	45.8%	42.4%	56.5%	<0.001
SLIT3	5.0%	11.8%	8.3%	18.2%	60.9%	<0.001
PCDHG	18.3%	47.1%	33.3%	48.5%	73.9%	<0.001
ANKRD13B	11.7%	35.3%	45.8%	30.3%	65.2%	<0.001
ADGRB1	28.3%	52.9%	33.3%	36.4%	65.2%	<0.001
THBD	21.7%	29.4%	33.3%	18.2%	82.6%	<0.001
C9orf50	15.0%	47.1%	8.3%	21.2%	65.2%	<0.001
